# Supplementary material for: Cryo-EM of full-length α-synuclein reveals fibril polymorphs with a common structural kernel
Source: Nat Commun. 2018 Sep 6;9:3609. doi: 10.1038/s41467-018-05971-2 (PMC6127345; doi:10.1038/s41467-018-05971-2)
Supplement: Supplementary file 1 — Supplementary Information [file 41467_2018_5971_MOESM1_ESM.pdf]

Supplementary Information

for

Cryo-EM of full-length α-synuclein reveals fibril polymorphs with a common structural kernel

B. Li, P. Ge & K. A. Murray. *et al*.

a

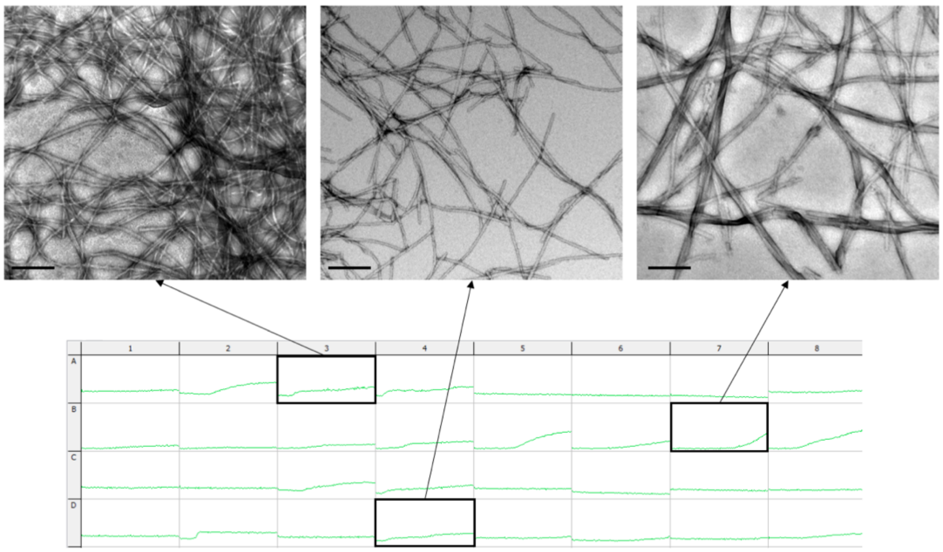

Supplementary Figure 1

Various fibril growth conditions yielded a range of human aSyn fibril polymorphs

The aggregation kinetics of the full-length human aSyn fibril growth were monitored by thioflavin T assay in various pH, salts, and additives in quiescent conditions for more than 14 days (fibril growth conditions described in Methods). The fibril morphology was subsequently characterized using negative-stain EM. Lower panels show example plots of ThT kinetics data. Upper panels show selected EM images from ThT-positive conditions, displaying a variety of fibril morphologies. Out of hundreds of fibril growth conditions screened, one fibril preparation (300 µM aSyn, 15mM tetrabutylphosphonium bromide, room temperature) with well-separated single filaments (Supplementary Fig. 2) was selected for further characterization. Scale bar: 200 nm.

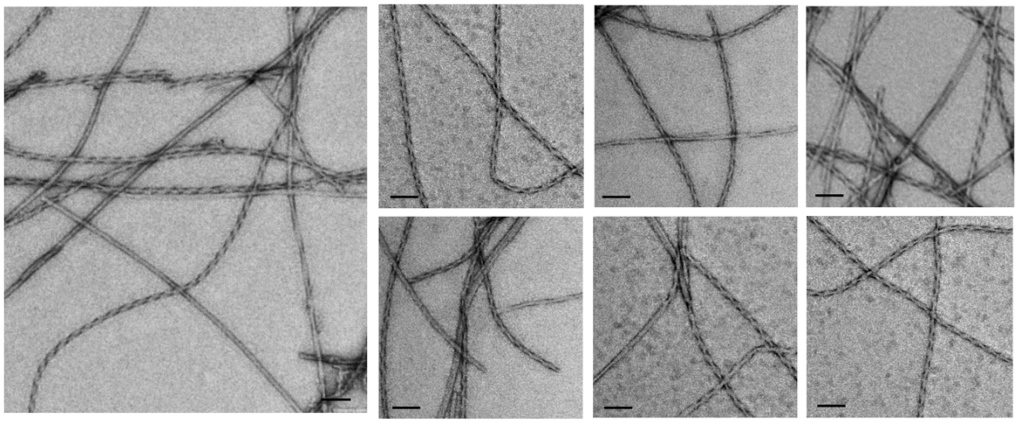

Supplementary Figure 2

Additional negative stain EM images of the aSyn fibril preparation selected for further characterization.

The fibril preparation (300 µM aSyn, 15mM tetrabutylphosphonium bromide, room temperature) was used for structural and biochemical characterization for the rest of our study. This fibril preparation displayed well-separated twisted or non-twisted filaments, where the rod and twister polymorphs are the two major populations. Scale bar: 100 nm.

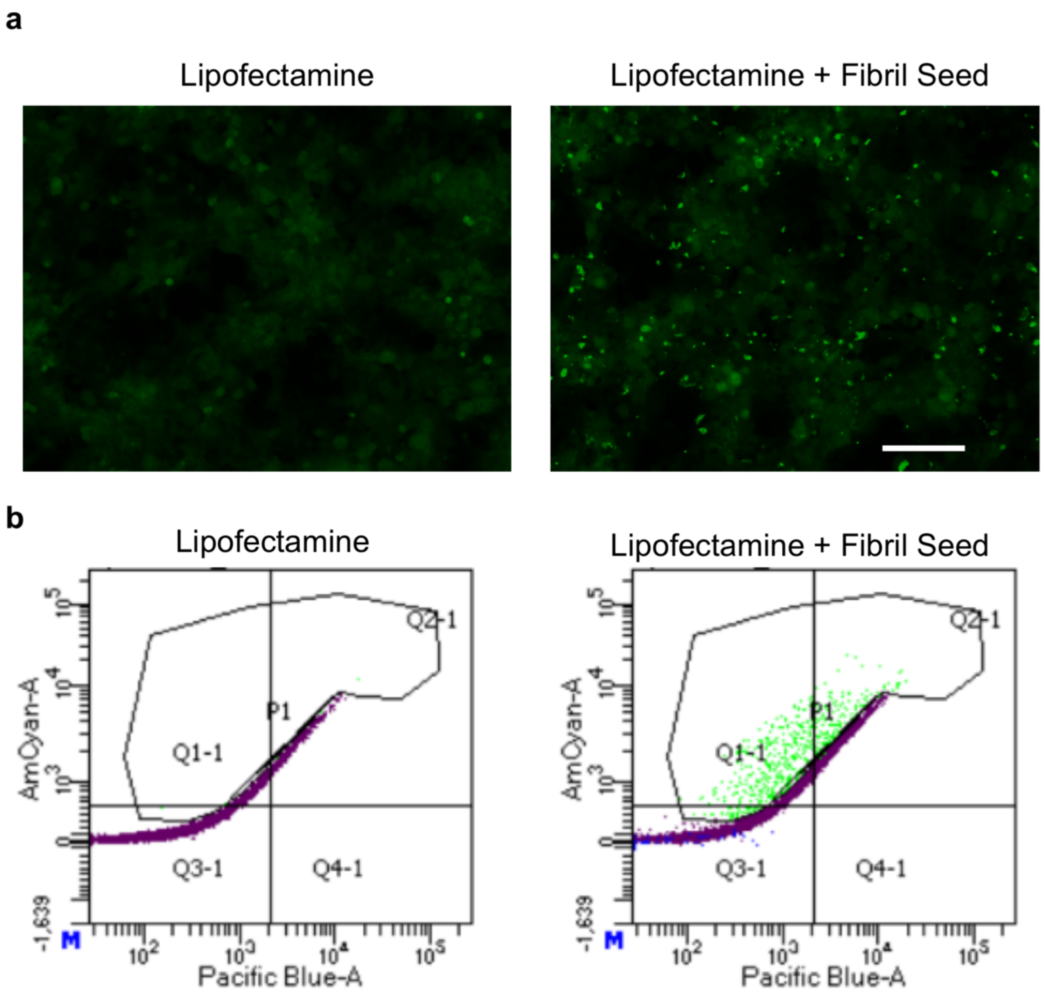

Supplementary Figure 3

Selection criteria for FRET analysis of seeded aSyn aggregation

(**a**) Transduction of 100 nM fibrils induced cellular aSyn aggregation as indicated by fluorescent puncta (right panel) in the aSyn biosensor cells[1]. Fluorescent images of aSyn Biosensor cells obtained 48 hours after treatment of Lipofectamine only (left) or with sonicated fibrils (right). Scale bar: 100 µm. (**b**) Gating used for flow cytometry-based FRET analysis to quantify intracellular aSyn aggregation. A polygon gate include the FRET-positive cells (green dots, aSyn aggregation or inclusions in cells) treated with fibril seeds and exclude the FRET-negative cells (purple dots, non-aggregated aSyn in cells) treated with only Lipofectamine.

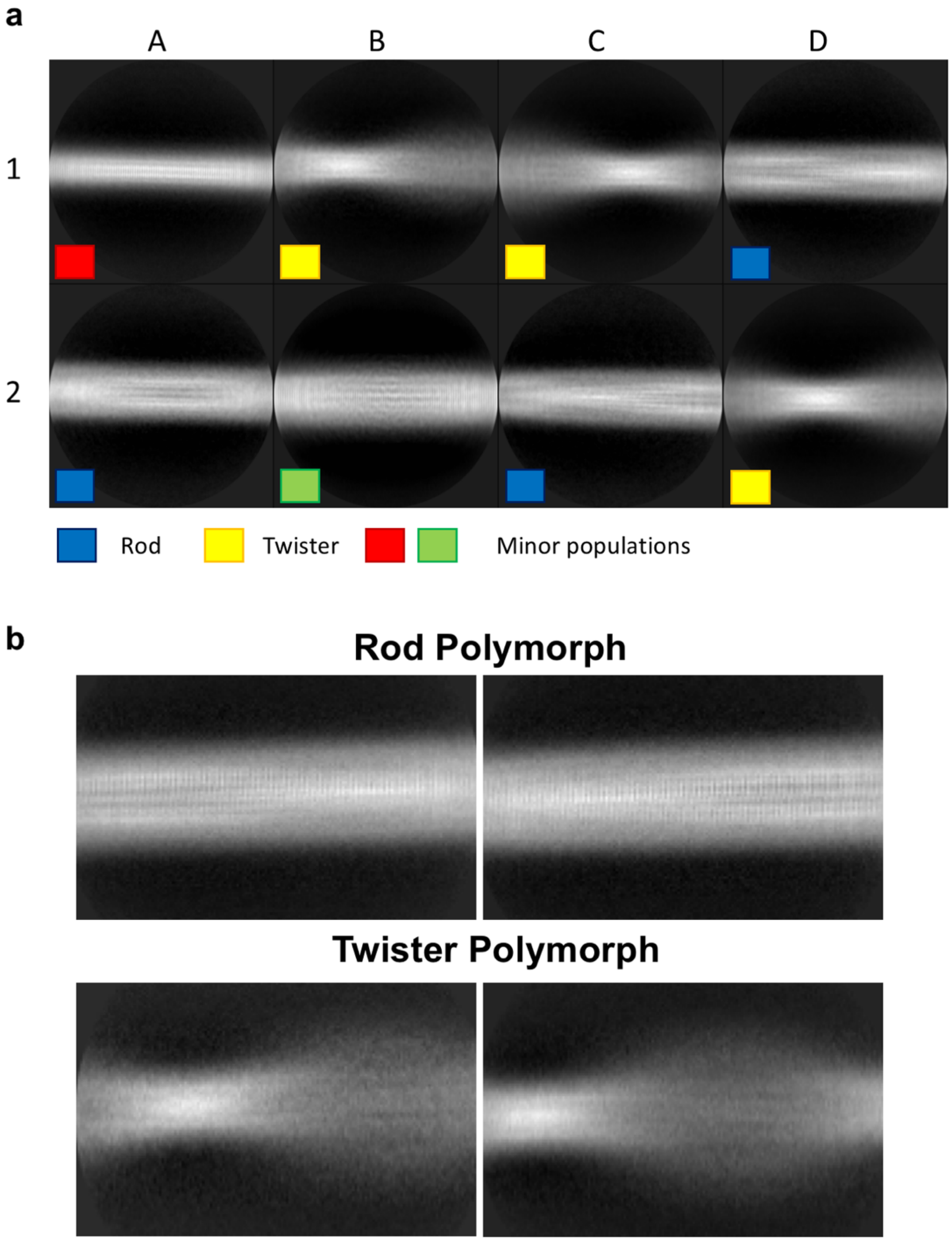

Supplementary Figure 4

2D class-averages for all extracted particles from cryo-EM images of the aSyn fibrils.

(**a**) Representatives of 2D class-averages for the two major (yellow and blue) and two minor (red and green) fibril populations. (**b**) Enlarged views of the rod and twister polymorphs to show details of the β-strand characteristic of a fibril.

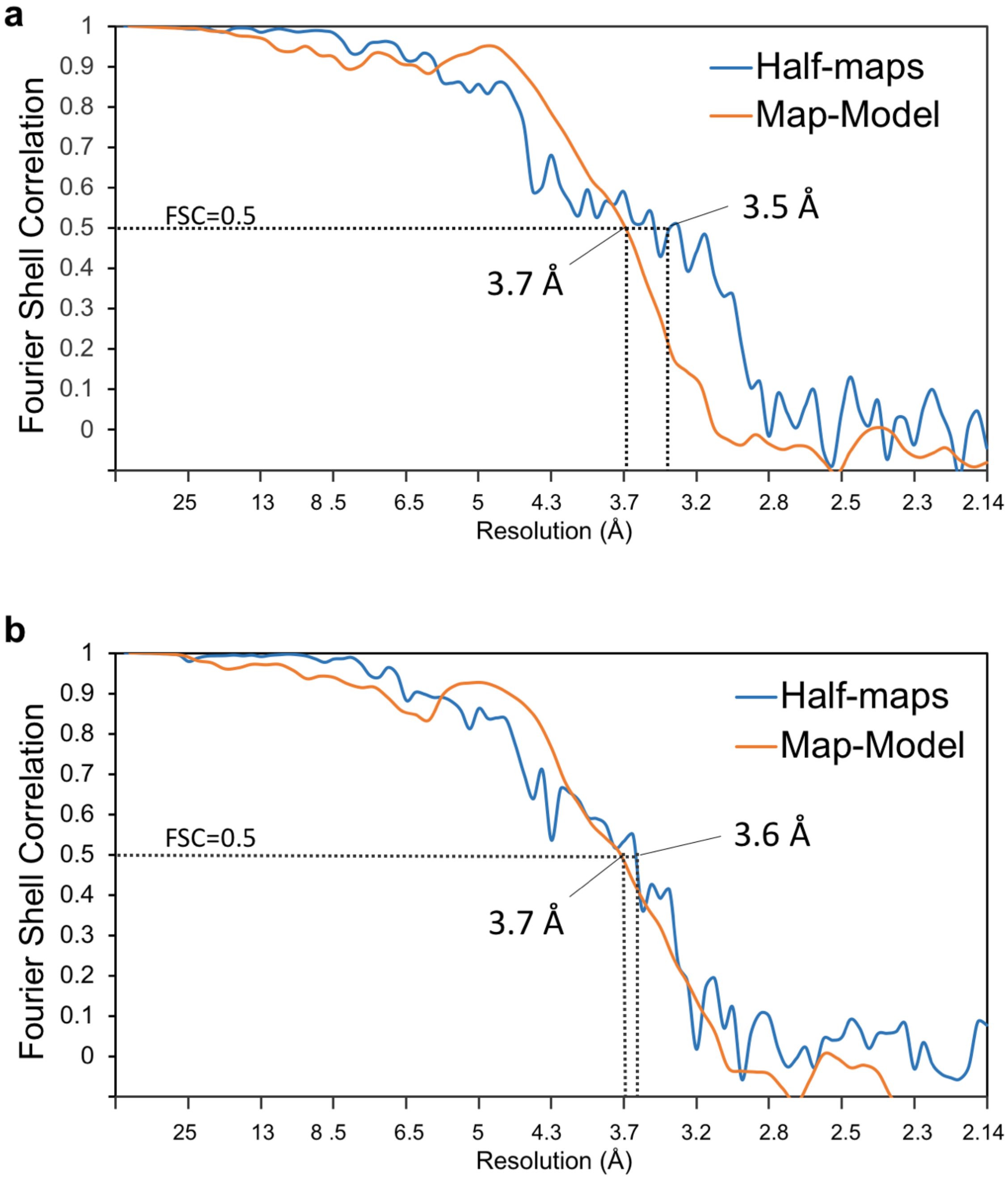

Supplementary Figure 5

Resolution estimation of the two cryo-EM maps by Fourier Shell Correlation (FSC)

An FSC cutoff value of 0.5 results in the resolutions of 3.5 Å for the half-maps and 3.7 Å for the map-model correlations for the rod polymorph (**a**) and 3.6 Å for the half-maps and 3.7 Å for the map-model correlations for the twister polymorph (**b**).

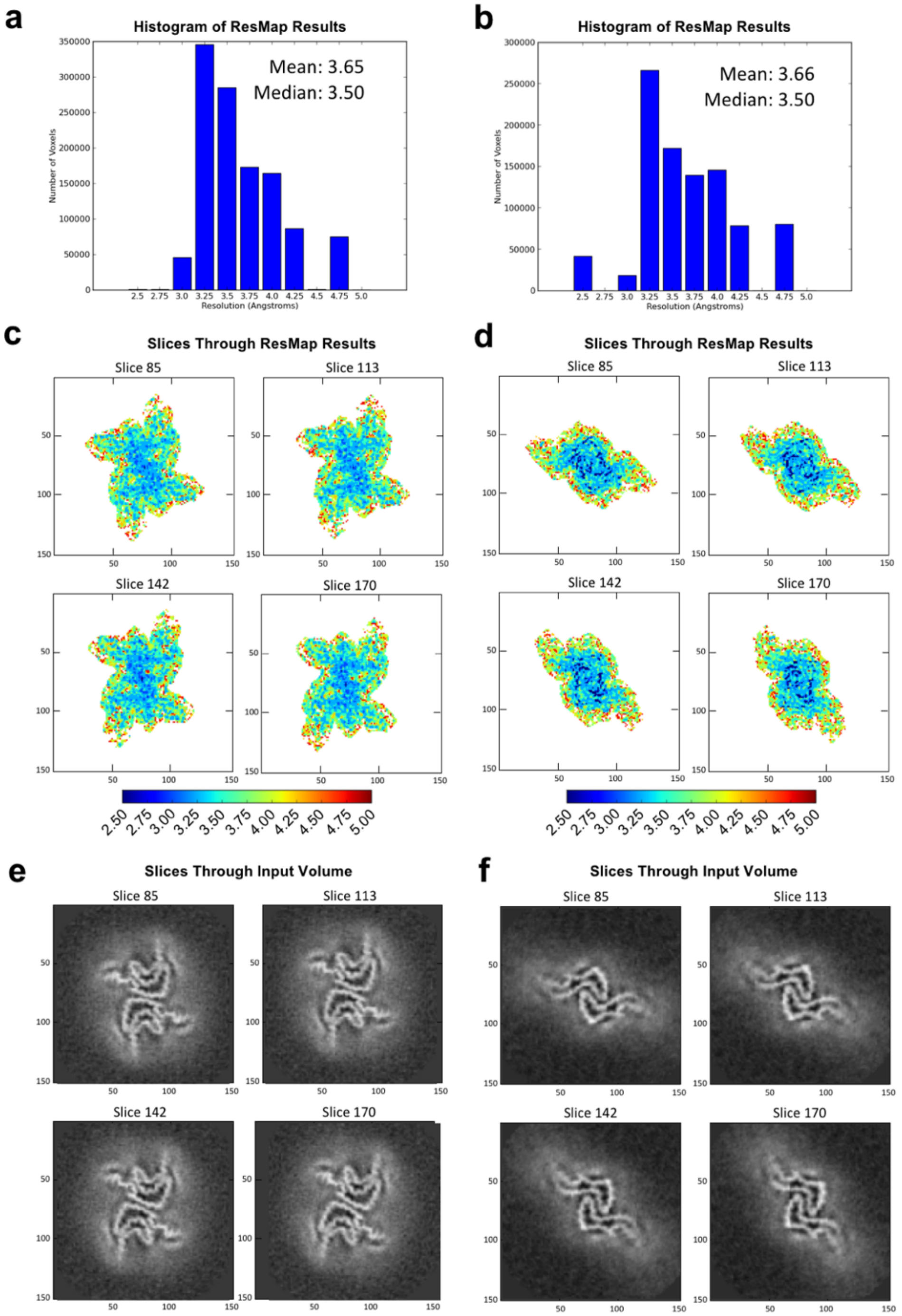

Supplementary Figure 6

Local resolution estimations for the rod (a, c and e) and twister (b, d and f) polymorphs showing good local resolution in the ordered fibril core region.

(**a** and **b)** Mean local resolutions for pixels within the mask for the rod polymorph is at 3.65 Å, and that for the twister polymorph is at 3.66 Å. (**c** and **d**) ResMap (Resolution Map) slices, perpendicular to the primary fibril axis, highlight good local resolutions (blue, 2.5 Å to 3.0 Å) at the central region of fibrils. (**e** and **f**) Density slices reveal a clear backbone for each fibril polymorph.

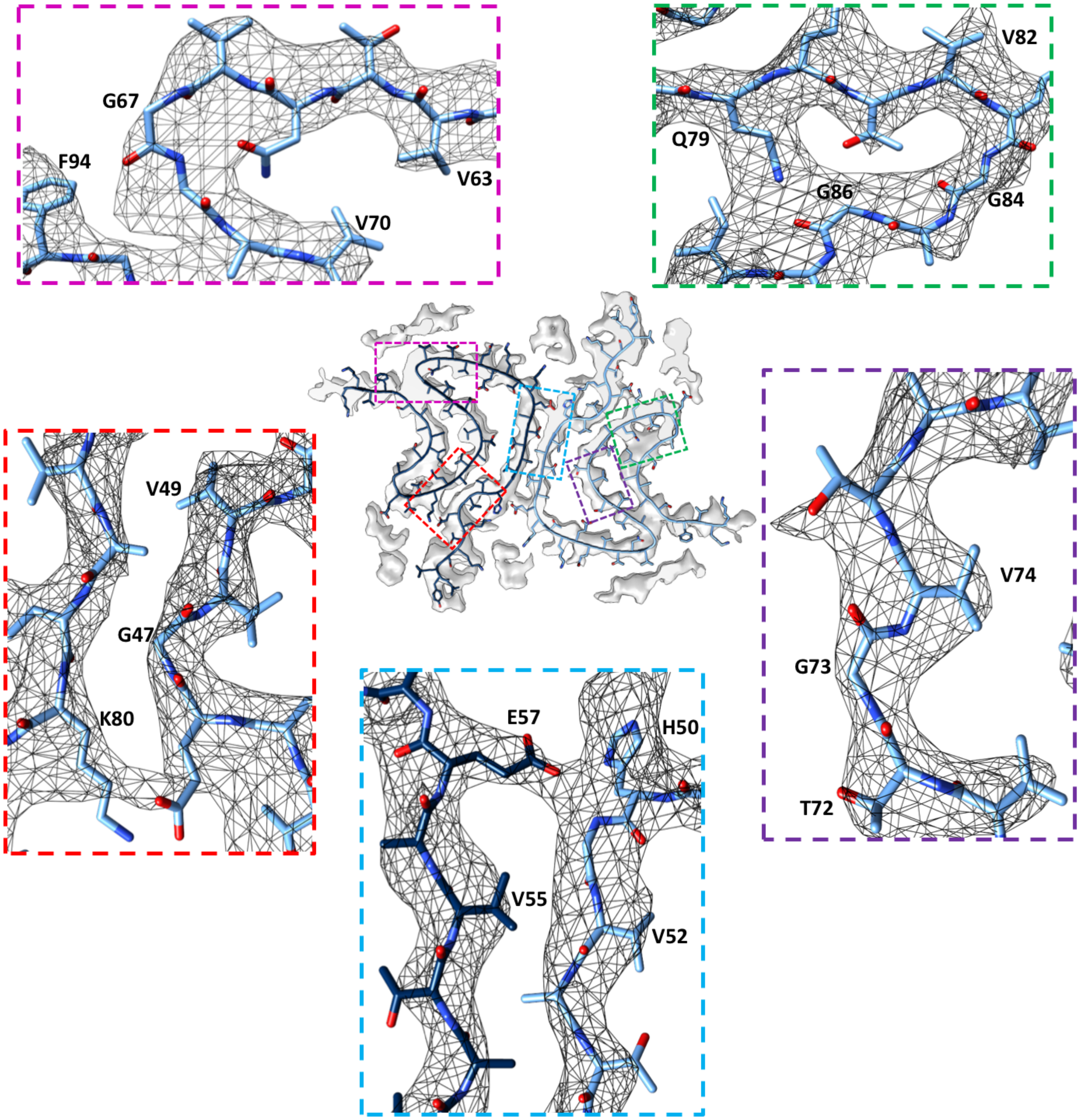

Supplementary Figure 7

Atomic model of aSyn rod polymorph structure matches well with its cryo-EM map

The atomic model shown in sticks was fit to the density map (shown in the gray chicken wire) using specific density landmarks. Large side chain densities were observed for aromatic residues (e.g. H50), and longer chain residues (e.g. E57, Q79). Areas showing reduced electron density for side chain were used to fit smaller residues (e.g. G47, G67, G73).

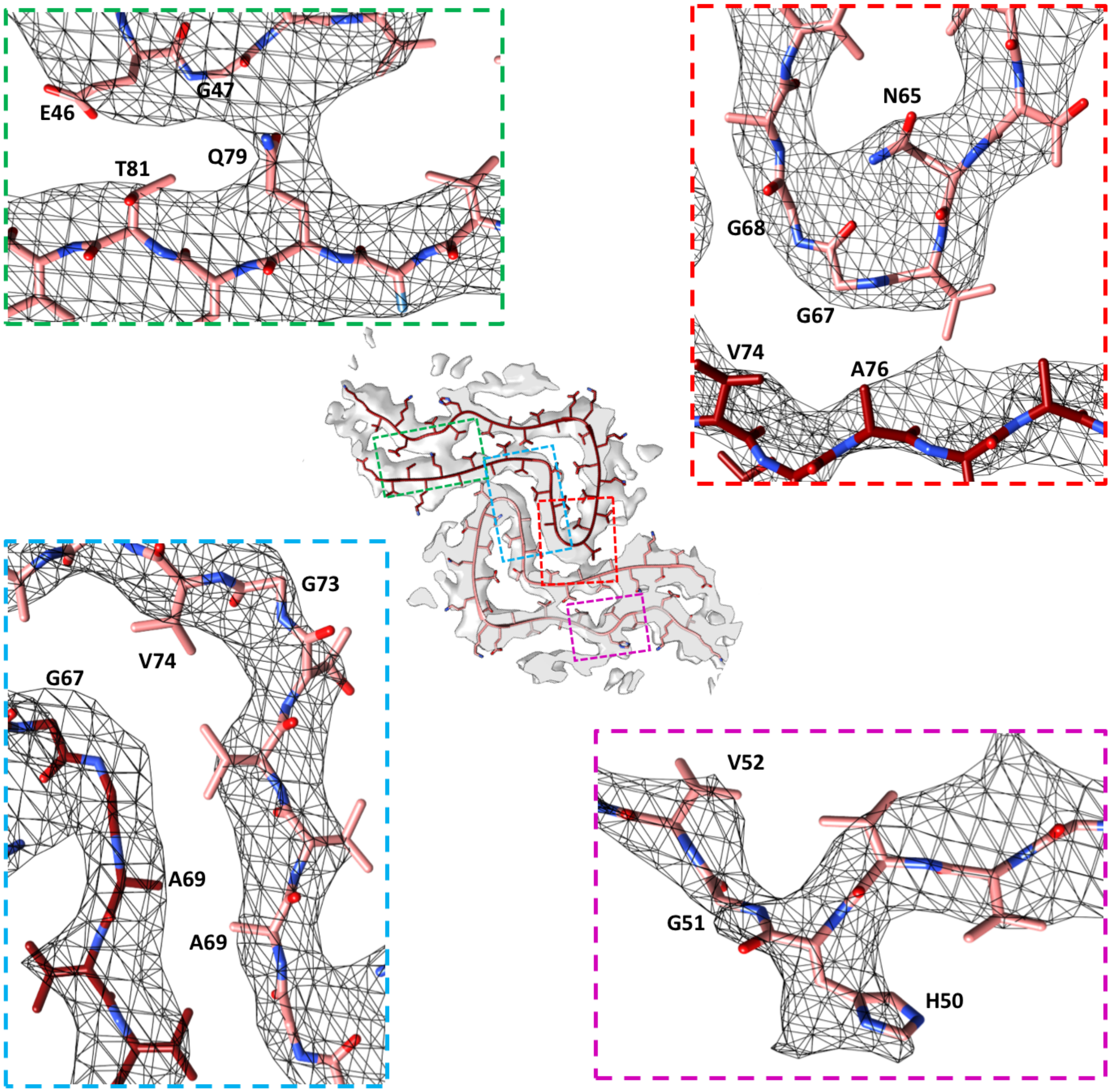

Supplementary Figure 8

Atomic model of aSyn Twister polymorph structure agrees well with its cryo-EM map

Key sidechain densities were used as landmarks and were utilized to determine the correct placement of the model. Aromatic (e.g. H50), and large charged residues (e.g. Q79) had large obvious densities branching from the backbone density.

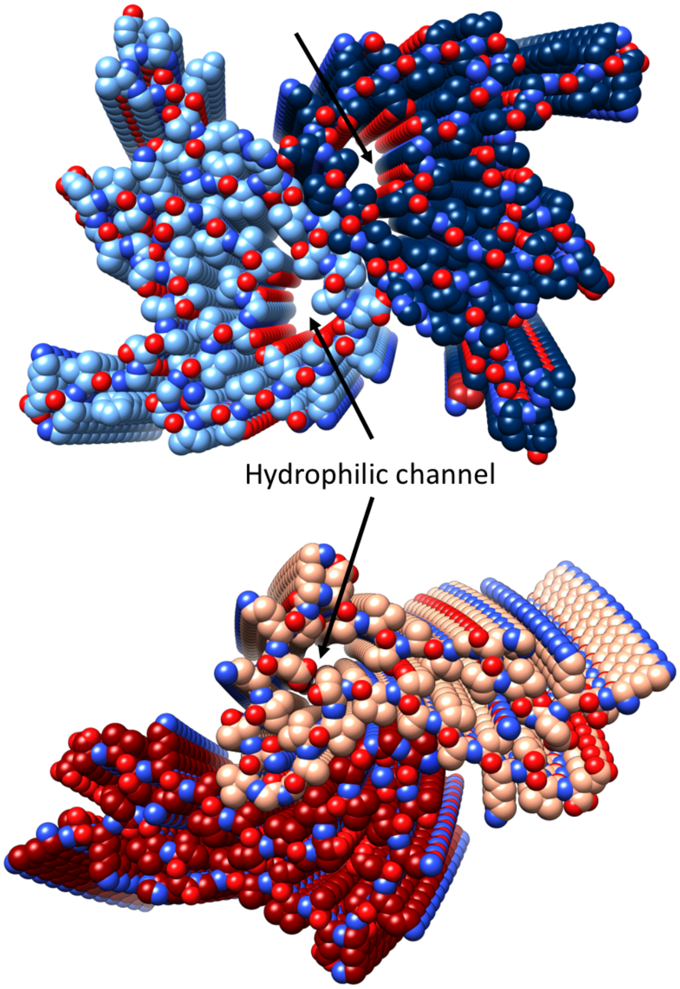

Supplementary Figure 9

Hydrophilic channels in the rod and twister structures

A hydrophilic channel, surrounded by residues T54, T59, E61, T72, and T75, locates next to the fibril core of either the rod (top) or twister (bottom) structure.

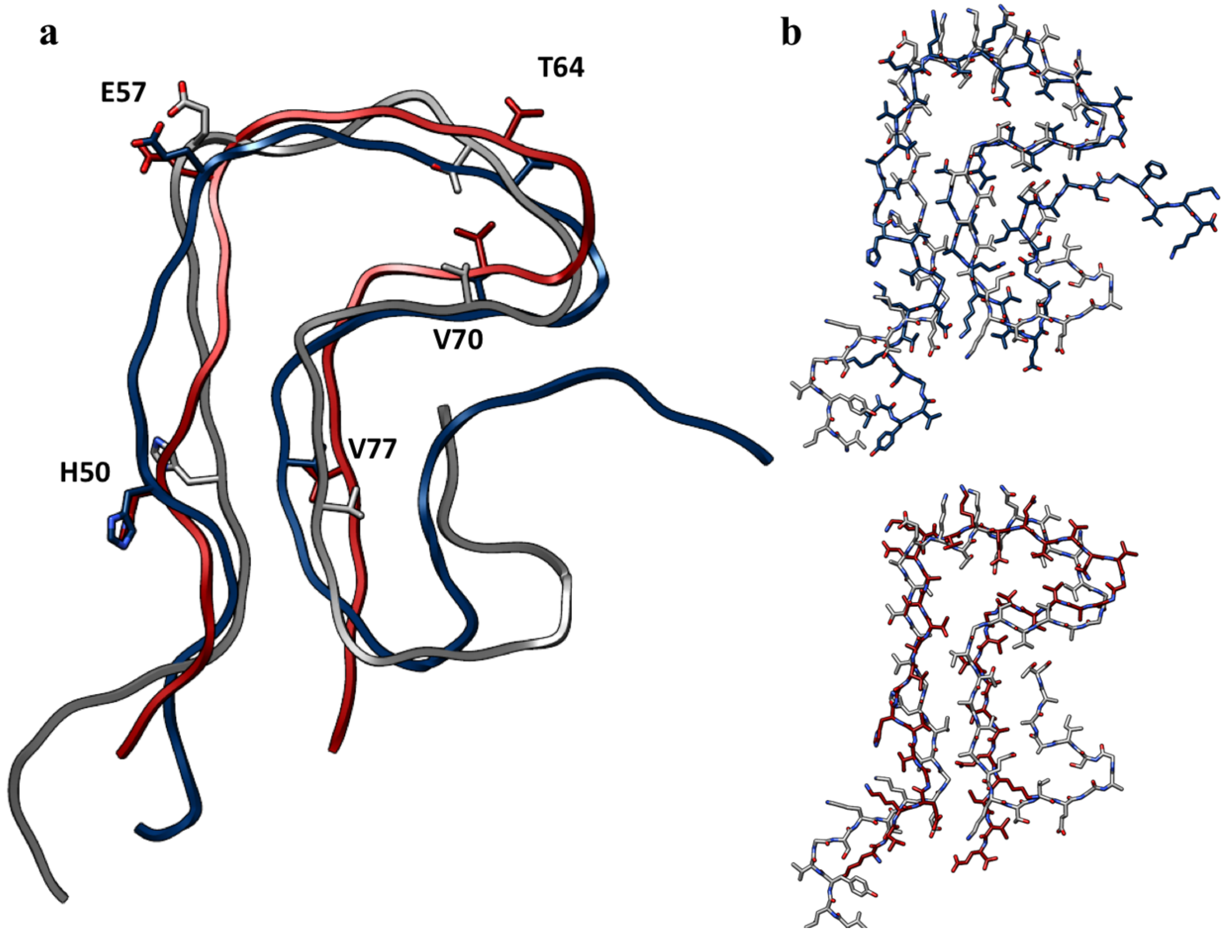

Supplementary Figure 10

Overlay of the rod and the twister protofilaments with the ssNMR protofilament

(**a**) Backbone overlay highlights resemblance in the structures between the ssNMR protofilament (gray) and the rod polymorph (blue) (Cα RMSD: 3.4 Å), ssNMR and the twister porlymorph (red) (Cα RMSD: 3.4 Å) in the 28 matched residues. (**b**) Full atomic models of the ssNMR compared with the rod polymorph (top) and the twister polymorph (bottom).

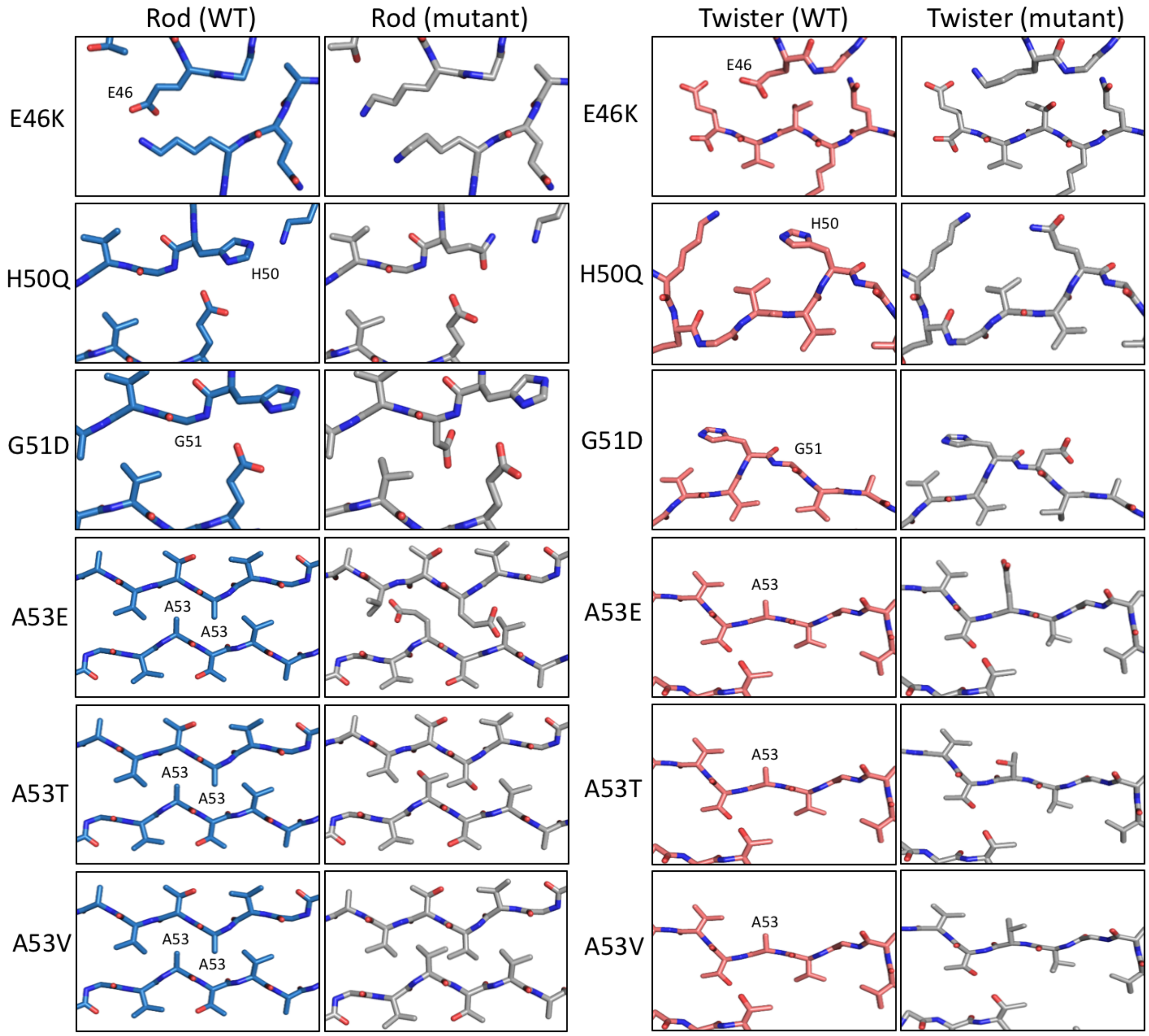

Supplementary Figure 11

Modeling of the familial mutations indicating the interruption of zipper interfaces in the rod structure

Viewed down the fibril axis, structural models of six PD familial mutations sites (E46K, H50Q, G51D, A53E, A53T and A53V) are compared with those of wild type residues in either rod (left) or twister (right) structures. The mutations are modeled upon the cryo-EM structures, and one strand from each protofilament is shown to highlight the modeled interactions. Those mutants are predicted to undermine stability in the preNAC steric zipper in the rod polymorph. On the contrary, the mutations sites in the twister structure located far from the fibril core which has little effect to the fibril core.

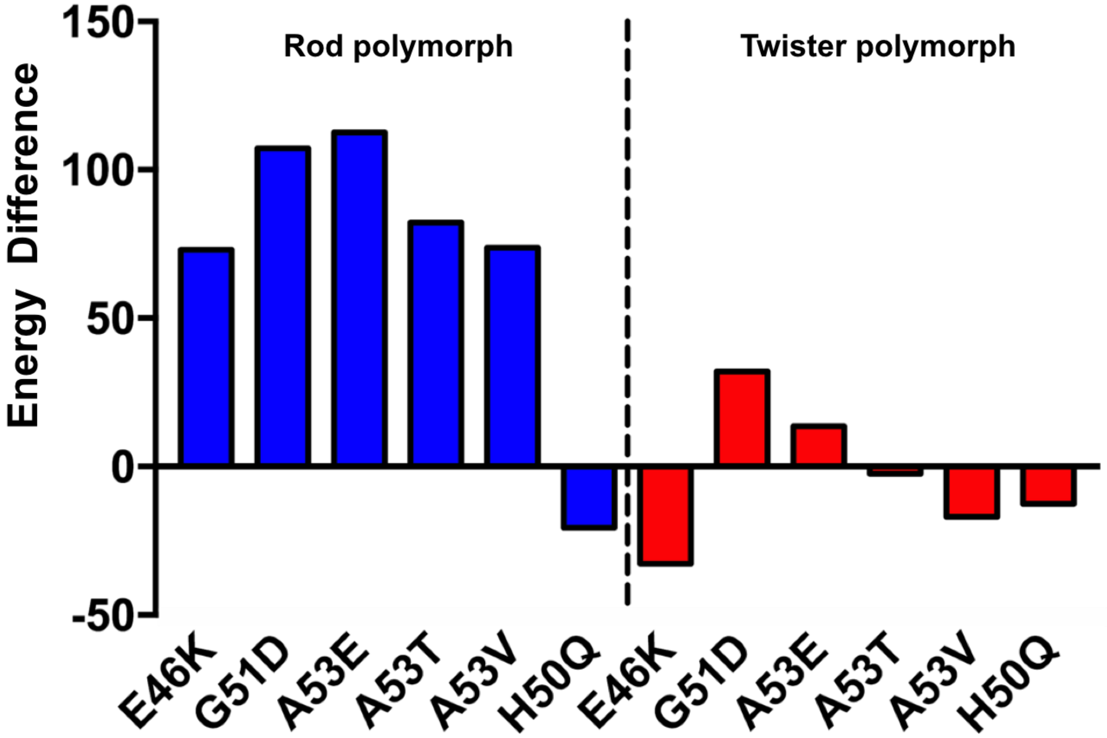

Supplementary Figure 12

Familial mutations (E46K, G51D, A53E, A53T and A53V) destabilize the rod structure (blue) while have minor impact in the twister structure (red).

Energies for the wild type and familial mutant were calculated using Rosetta[2]. The contribution of each mutant was evaluated by the score difference between the mutant and the WT. When comparing the energy evaluation in both rod and twister structures, familial mutations E46K, G51D, A53E, A53T and A53V have greater energy differences (>50 Rosetta energy units) in the rod structure, indicating most of mutants may disrupt the stability of the fibril core. Smaller energy difference (within ±30 Rosetta energy units) is observed in the twister structure.

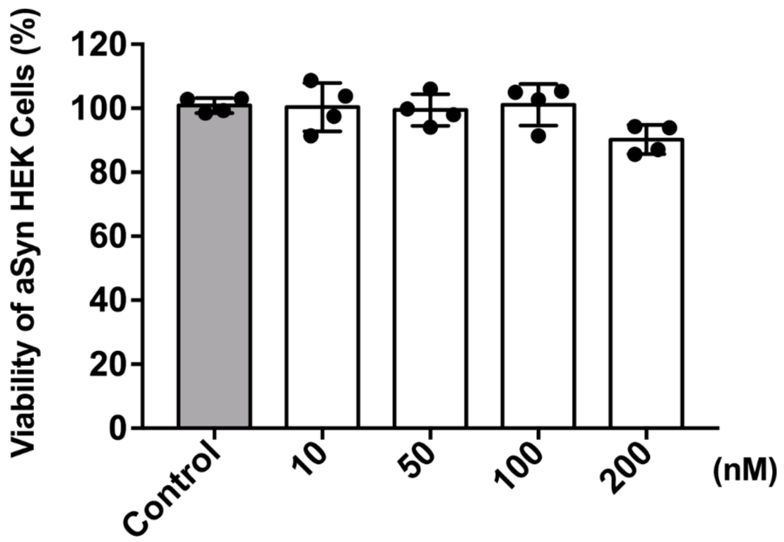

Supplementary Figure 13

Sonicated aSyn fibrils at different tested concentrations showed no significant toxicity in HEK biosensor cells

Cytotoxicity of aSyn fibrils is measured using MTT-based cell viability assay of aSyn biosensor HEK293T cells. The aSyn fibrils have shown no significant cytotoxicity at the tested concentrations up to 200 nM ($p \geq 0.05$ vs. control). Data are presented as mean ± standard error.

Supplementary References

1. Prusiner, S. B. *et al.* Evidence for α-synuclein prions causing multiple system atrophy in humans with parkinsonism. *Proc. Natl. Acad. Sci.* **112,** E5308–E5317 (2015).

2. Leaver-Fay, A. *et al.* Rosetta3: An object-oriented software suite for the simulation and design of macromolecules. *Methods in enzymology* **487,** 545–574 (2011).
